# Strengthening integrated community case management through digitalization and performance management in Busia County, Kenya

**DOI:** 10.3389/fpubh.2025.1612973

**Published:** 2025-09-03

**Authors:** Joseph Kyalo Njoroge, Tabither Muthoni Gitau, Erick Kiprotich Yegon, Nzomo Mwita, Alice Koimur, Marlyn Ochieng, Rhonnie Omollo, Rosebellah Amihanda

**Affiliations:** Living Goods, Nairobi, Kenya

**Keywords:** iCCM, performance management, digitalization, CHWs, Busia, Kenya, care-seeking behavior

## Abstract

Kenya continues to face a high burden of childhood mortality driven by preventable illnesses such as diarrhea, malaria, and pneumonia. Integrated community case management (iCCM) offers a strategy for mitigating this burden through community-level diagnosis, treatment, and referral. This study assessed the role of digitalization and performance management in enhancing the effectiveness of the iCCM strategy in Busia County. Using a quasi-experimental design, a comparison of iCCM intervention outcomes in Nambale sub-county with a non-iCCM comparison site in Teso South sub-county was done. Data were collected through household surveys and in-depth interviews. Results showed a significant improvement in healthcare-seeking behavior in the intervention site, with 56% of caregivers reporting that they sought care for pneumonia on the same day of symptom onset, compared to 35% in the comparison site (*p* = 0.031). Additionally, findings indicate that CHPs were a preferred point of care for iCCM cases in the intervention site, managing 38% of malaria cases, 19% of pneumonia cases, and 25% of diarrhea cases at the community level. This contrasts with the comparison site, where only 16, 3.7, and 0% of malaria, pneumonia, and diarrhea cases, respectively, were managed by CHPs, all differences being statistically significant (*p* < 0.05). The significantly higher proportion of iCCM cases managed by CHPs in the intervention site was associated with a lower reliance on facility-based care. Only 42% of ARI cases were managed at government facilities in the intervention site compared to 62% in the comparison site (*p* = 0.024), while just 1.9% of cases were handled at private facilities in the intervention site versus 12.2% in the comparison site (*p* = 0.03). These improvements were made possible by the integration of the digital tools and robust performance management practices. This combination enhanced CHWs effectiveness, strengthened health seeking behaviors, and contributed to the overall success of the iCCM strategy in the intervention area. In conclusion, digitalization and robust performance management strengthen the iCCM strategy leading to improved by improving health care seeking for childhood illnesses behaviors and reduce health system burden in low-resource settings.

## 1 Introduction

Sub-Saharan Africa (SSA) continues to experience high mortality among children under five, primarily due to preventable diseases such as diarrhea, malaria, and pneumonia, with malnutrition as an underlying factor (1). In Kenya, under-five mortality has declined by 50% from 102 per 1,000 live births in 1999 to 53 per 1,000 in 2022 (2). Despite this progress, Kenya fell short of the Millennium Development Goals (MDGs) and now faces pressure to meet Sustainable Development Goal (SDG) targets by 2030 (3).

Kenya introduced the Integrated Management of Childhood Illnesses (IMCI) strategy in 1999; however, inadequate coverage, poor access, underutilization of guidelines, and lack of funding undermined its effectiveness, particularly the community component (4). The iCCM framework launched in 2015 under Kenya’s Community Health Strategy (CHS) targets common child illnesses through CHWs, especially in hard-to-reach areas (5). The framework is a platform for community management of childhood diarrhea, malaria, pneumonia, neonatal illness, and malnutrition (6). The framework is anchored in the CHS (7) and the Child Survival and Development Strategy (8), which address key areas including policy, management of cases and the commodity supply chain, and supervision. Within the national iCCM implementation framework, guidelines exist on training for CHWs to treat children aged below 5 years with diarrhea using oral rehydration salts (ORS) and zinc, to diagnose malaria with a malaria rapid diagnostic test (mRDT) and treat it with artemisinin combination therapy (ACT), and to refer suspected pneumonia, mild to moderate malnutrition, and sick newborn to a health facility (MoH, iCCM participants manual). This is in line with the components of iCCM as recommended by WHO and UNICEF. It also includes referrals of the newborn delivered at home and any newborn or mother with danger signs (9). The implementation is done by CHWs at household and/or community levels.

This strategy aimed at training, supporting, and equipping CHWs to deliver diagnostic, treatment, and referral services for three major childhood illnesses: malaria, pneumonia, and diarrhea. The CHWs were digitalized with mobile phones that had a digital health information application that not only facilitates reporting of the services delivered but also drives greater quality health care to the community. This reporting facilitated clear visibility of the coverage and timeliness of service delivery from the CHWs’ end but also enabling real-time monitoring from the supervisor’s application thereby facilitating evidence-based performance management. However, large-scale implementation has yielded mixed results, leading to limited coverage and minimal service integration. Additionally, the impact of large-scale iCCM programs on reducing child mortality has not been conclusively demonstrated. Therefore, this study was designed to assess the contribution of the iCCM project in increasing coverage and appropriate treatment of malaria, diarrhea, and pneumonia in children under 2 years of age, as well as the coverage of other key maternal and child health interventions.

Living Goods has partnered with Kenya’s Ministry of Health and county governments to strengthen community-based primary healthcare and advance universal health coverage. In Busia County, they implement the DESC approach- Digitally enabled, Equipped, Supervised, and Compensated—to deliver high-quality, cost-effective community health services. This model empowers community health workers (CHWs) with digital tools, supplies, supervision, and fair compensation, enhancing their performance and strengthening community health systems. Previous Living Goods initiatives demonstrate that DESC improves CHW motivation, accountability, and service quality, leading to better health outcomes in low-resource settings (10).

This study presents findings from the implementation of the integrated community case management (iCCM) intervention in Busia County, Kenya. It examines the impact on care seeking, treatment, and coverage for malaria, pneumonia, and diarrhea among children under five, at community level by CHWs. The intervention incorporated the DESC approach used by Living Goods, which ensures that Community Health Workers (CHWs) are digitally enabled, equipped with essential supplies, supervised (by supervisors utilizing the performance management approach) and compensated.

In the intervention site, Nambale, CHWs known as Community Health Promoters (CHPs) delivered iCCM using a mobile smart health application. This application aligned with guidelines from the (11). The tool guided CHPs through standardized assessment protocols based on presenting symptoms such as cough, fever, or diarrhea. It helped identify iCCM entry points, supported illness classification, and enabled appropriate treatment and/or referral. The application promoted protocol-driven decisions by prompting critical tasks such as timed breath counts and providing feedback to guide diagnosis based on the child’s age. It also included structured assessments for malaria and diarrhea, and embedded checks for danger signs to reduce the risk of missed referrals. In addition to digital case management, CHPs were supported through real time dashboards that allowed supervisors to track performance and offer timely coaching. CHPs also received refresher training and participated in a performance-based incentive model, which contributed to greater motivation, consistency in service delivery, and improved data quality.

In contrast, CHPs in the comparison site, Teso South, followed the same national guidelines but used paper tools for assessments. They mainly focused on community case management hence assessing and managing Malaria cases alone and referring all diarrhea and Pneumonia cases identified in the community. Further, they lacked access to digital guidance or real time performance monitoring, which may have affected the accuracy, timeliness, and consistency of illness classification and referrals.

## 2 Materials and methods

### 2.1 Study design and setting

This was a quasi-experimental study design adopting a static-group comparison approach. In this form the outcomes of interest were measured once, following exposure of a non-random group of participants to an intervention (Harvey and Kent, 2018), and matched to a comparison. Mixed methods approach was used for data collection. The intervention effect was determined in the two sub-counties, one with iCCM and the other without iCCM being implemented. Nambale sub-county served as the intervention site, where iCCM was implemented, while Teso South served as the comparison site, with no recent iCCM activities.

### 2.2 Sample size determination

An iCCM coverage rate of 50% was assumed, informed by findings from a South African study that reported under-five household coverage by community health workers ranging between 30 and 90%. The midpoint estimate of 50% was adopted as a conservative assumption for modeling purposes (12). The evaluation hypothesized that non-iCCM community units would exhibit a 20 percentage point higher prevalence of childhood illness (malaria, pneumonia, or diarrhea) compared to iCCM sites. To detect this difference with 90% power and a 5% level of significance, the required sample size per study group was determined using the formula for comparing two proportions, as described by Fleiss with continuity correction (13). The initial calculation yielded a minimum sample of 125 participants per group. This was then adjusted to account for a design effect of 2.0, due to the cluster sampling design, and a 20% inflation factor to cater for non-response and refusals. The final adjusted sample size was 400 participants per group, resulting in a total sample of 800 participants across both intervention and control sites.

Qualitative sample size was determined using theoretical saturation cap as described by Francis et al. (14). The study targeted a minimum of two in-depth interviews (IDIs) with each respondent group, Community Health Promoters (CHPs) and Women of Reproductive Age (WRAs), per Community Health Unit (CHU). This approach aimed at capturing both the provider and client perspectives across all study sites. In total, this yielded a target of 64 IDIs: 32 with CHPs and 32 with WRAs, ensuring diverse voices and experiences were represented.

### 2.3 Sampling

**Figure 1:**
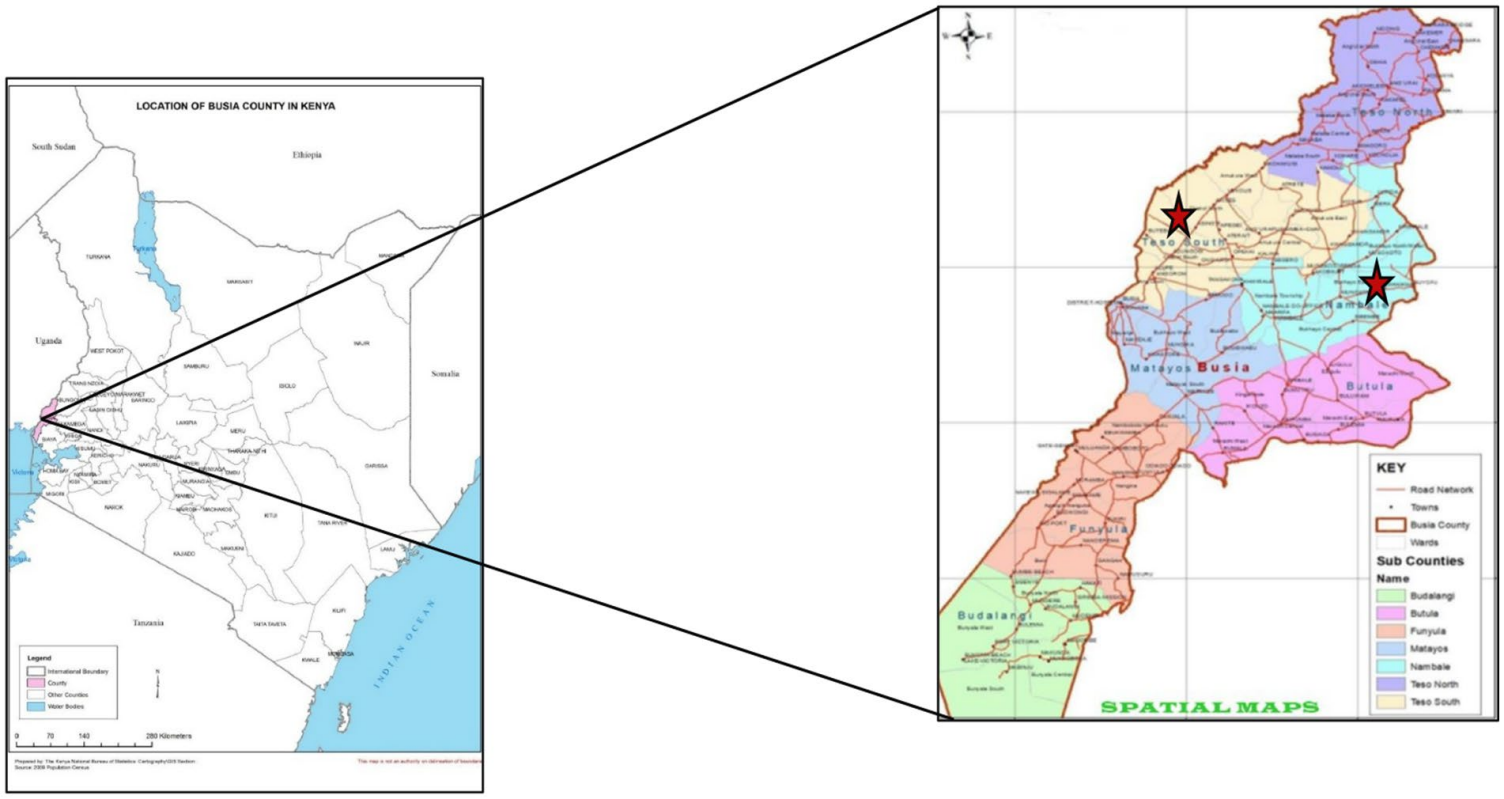
Administrative Units of Busia County in Kenya.

The study was conducted in two locations within Busia County. Nambale sub-county served as the site for the integrated Community Case Management (iCCM) intervention, while Teso South sub-county acted as the comparison site. The population of Nambale sub county was 111,636, while that of Teso South sub county was 113,097 (15). The intervention site was purposely selected because it has been implementing iCCM intervention. Living Goods has been implementing iCCM in Nambale sub county since 2017 with the Busia County health department. On the other hand, the comparison site was selected since it has no iCCM interventions. Additionally, it has comparable socioeconomic and demographic profile to the intervention site. These included level of education, marital status, fuel used mainly for household cooking, main source of drinking water and toilet facilities in the community. The comparison site has community health units where CHWs were trained in the Ministry of Health basic CHW package but not iCCM (Figure 1).

A two-stage household cluster sampling was used in both intervention and comparison groups. Community Health Units (CHUs) were selected as clusters, with 8 CHUs in the intervention site and four in the comparison site. CHU is the foundational tier for delivering health services at the community level. It is formally identified as the first level of health services provision within the national health system and typically comprising 1,000–5,000 people, served by CHWs under the Community Health Strategy. Each CHP had 45 households in the intervention and 90 households in the comparison group. To create a sampling frame, household information was collected, including the household head’s name, the number of women of reproductive age (WRA), WRAs’ ages, and ages of children. This data helped identify eligible households that had children under five who had been residents of the study site for at least 6 months. In the comparison site, CHWs updated household listings using a template, while in the intervention site, data from the Smart Health application’s server was used. Untraceable households were replaced by the next household in the same village.

For qualitative sampling, respondent selection was purposive, guided by roles, experience, and relevance to the study objectives. The sampling was conducted to the point of thematic saturation, where additional interviews no longer revealed new insights. This strategy enabled the study to gather rich, context-specific data that reflected the breadth and depth of experiences related to community health service delivery, performance, and user satisfaction. Efforts were made to ensure diversity in geographic location, facility catchment area, and performance level of CHUs to enhance the credibility and transferability of the findings. After conducting three to interviews we assessed whether new information was still emerging; additional interviews were held until no new themes appeared.

### 2.4 Data collection

Data collection commenced on Wednesday 14th – and continued up to 23rd December 2022 in both the intervention and comparison sites simultaneously. Quantitative data were collected via structured household surveys targeting primary caregivers of children under five. Quantitative data from structured questionnaires were collected using mobile phones with SurveyCTO software, and data were transmitted to Aggregate Server immediately after collection for quality review by the study team.

Qualitative data were obtained through in-depth interviews and FGDs with CHWs and key stakeholders. Interview and focus group discussion (FGD) sessions were audio-recorded and subsequently transcribed in verbatim. Transcripts in the original language (Kiswahili) were reviewed by the lead author. Interview transcripts were translated into English independently by two translators. For FGDs, two separate transcripts were produced and compared for accuracy; discrepancies were resolved through joint review of the audio recordings by the lead author and enumerators, followed by necessary adjustments.

Quantitative analysis was performed in SPSS, utilizing independent t-test to assess difference in means between the intervention and comparison groups at *p* < 0.05. Qualitative data was analyzed using thematic analysis. A deductive approach was applied to closely examine the data and identify common themes and recurring patterns of meaning. Atypical responses were also noted and documented as either divergent views or entirely new themes.

### 2.5 Ethics statement

Ethical approval was obtained from the Kenya Medical Research Institute (KEMRI SERU) and the National Commission for Science, Technology and Innovation (NACOSTI). Informed consent was obtained from all participants through formal meetings and information sheets. Participation was voluntary, with the option to refuse without penalty. All data were anonymized to ensure confidentiality.

## 3 Results

### 3.1 Sample characteristics

Overall, the survey response rate was high, at 95.5% (382/400) in the intervention and 95% (380/400) in the comparison site, while for qualitative data the study engaged 32 CHPs and 38 WRAs in IDIs. The analysis revealed notable socio-demographic differences between Nambale and Teso South sub counties. Teso South had a significantly higher proportion of households in the lowest wealth quintile (24.5% vs. 15.2%, *p* = 0.008), indicating greater economic vulnerability. Additionally, more caregivers in Teso South lacked formal education compared to Nambale (4.2% vs. 1.6%, *p* = 0.007). Religious affiliation also varied, with a higher proportion of Roman Catholics in Teso South (33.2% vs. 25.7%, *p* = 0.028). Differences in occupation were evident, with farming more common in Teso South (60.5% vs. 49.7%) and self-employment more reported in Nambale (21.7% vs. 17.1%, *p* = 0.043). Lastly, household income sources differed significantly (*p* < 0.001), with Teso South more reliant on farming and produce sales, while Nambale reported higher engagement in small businesses. These differences provide important context for interpreting health behaviors and service access across the two areas as shown in Table 1.

**Table 1:** Demographic and socio-economic characteristics of household survey participants.

Variables		Total (*n* = 762)	Nambale (*n* = 382)	T. South (*n* = 380)	*p* value
Household wealth index in quintiles	First	19.8%	15.2%	24.5%	0.008
	Second	20.2%	19.1%	21.3%	
	Third	19.7%	21.2%	18.2%	
	Forth	20.3%	23.6%	17.1%	
	Fifth	19.9%	20.9%	18.9%	
Age categories of caregivers	Early reproductive age	30.2%	32.2%	28.2%	0.350
	Mid reproductive age	43.8%	43.7%	43.9%	
	Late reproductive age	26.0%	24.1%	27.9%	
Age categories of index child	Infancy	26.2%	25.1%	27.4%	0.597
	Toddler	21.4%	20.7%	22.1%	
	Early childhood	52.4%	54.2%	50.5%	
Caregiver level of school attended	None	2.9%	1.6%	4.2%	0.007
	Primary	60.1%	59.4%	60.8%	
	Secondary	29.9%	33.8%	26.1%	
	Tertiary	7.1%	5.2%	8.9%	
Religion	Roman catholic	29.4%	25.7%	33.2%	0.028
	Protestant	69.3%	72.3%	66.3%	
	Muslim	1.0%	1.8%	0.3%	
	African tradition	0.3%	0.3%	0.3%	
Caregiver occupation	Student	3.9%	3.7%	4.2%	0.043
	Formal employed	3.0%	2.6%	3.4%	
	Self employed	19.4%	21.7%	17.1%	
	Casual work	3.3%	4.2%	2.4%	
	Domestic Work	3.1%	3.4%	2.9%	
	Farmer	55.1%	49.7%	60.5%	
	Housewife	12.1%	14.7%	9.5%	
Household main source of income	Formal employment	8.0%	7.1%	8.9%	<0.001
	Small scale farming	25.7%	25.1%	26.3%	
	Farming/selling produce	26.0%	19.6%	32.4%	
	Small business/non-farm	21.9%	27.5%	16.3%	
	Petty trade	0.4%	0.3%	0.5%	
	Casual/informal labor	17.3%	19.6%	15.0%	
	Relatives/remittances	0.7%	0.8%	0.5%	

**Table 2:** Malaria: care-seeking and access to appropriate treatment.

Measure		Intervention [*n* = 382]	Comparison [*n* = 380]	*p*-value
Prevalence of malaria		24.9% (n = 95)	25.0% (n = 95)	0.966
Sought treatment [*n* = 95]		80.0%	71.6%	NS
Treatment source [*n* = 95]	Government facility	44.2%	49.5%	NS
	CHW	37.9%	16.8%	0.002*
	Pharmacy	17.9%	24.2%	NS
	Private facility	3.2%	10.5%	NS
	Home remedies	1.1%	2.1%	NS
Sought care [*n* = 95]	Within 24 h	58.9%	45.3%	0.309
	Following day	18.9	23.2%	
Tested/mRDT [*n* = 95]		74.7%	74.7%	-
Testing/mRDT source	Home by CHW	45.1%	16.9%	<0.001*
	Government facility	54.9%	71.8%	0.055
	Pharmacy	0.2%	12.7%	NS
Received anti-malarial treatment/ACT [*n* = 95]		84.2%	68.4%	0.027*
Malaria treatment source	Public Health facilities	40.0%	44.6%	NS
	Private hospitals	1.3%	15.4%	0.002*
	Pharmacy	24.5%	23.1%	NS
	CHW	35.0%	15.4%	0.008*
CHW follow-up on sick child [*n* = 95]		35.8%	16.8%	0.002*
CHW advice on how to prevent malaria [*n* = 95]		35.9%	17.9%	0.001*

*Significant at 0.05; NS” stands for “Not Significant” (*p* ≥ 0.05).

### 3.2 Integrated community case management outcomes

Despite comparable malaria prevalence (~25%) in both intervention (*n* = 382) and comparison (*n* = 380) sites, the intervention, centered on Community Health Workers (CHWs), significantly reshaped care-seeking and treatment access. While overall treatment seeking was similar (80.0% vs. 71.6%), the source differed markedly. CHW utilization for treatment was significantly higher in the intervention area (37.9% vs. 16.8%, *p* = 0.002), contrasting with a greater reliance on private facilities in the comparison group (10.5% vs. 3.2%, *p* = NS). Moreover, the study noted consistently lower proportion of patients having their first point of care at the health facilities at 47% in the intervention group compared to 59% in the comparison group with 41% being treated at the health facilities in the intervention group while 69% of the malaria patients received treatment at the health facilities. Although overall mRDT testing was equal (74.7%), CHWs facilitated significantly more home-based testing in the intervention area (45.1% vs. 16.9%, *p* < 0.001), while government facility testing was slightly higher in the comparison group (71.8% vs. 54.9%, *p* = 0.055).

The qualitative findings highlight the improvements in access to care for sick children as a contribution of CHWs implementing iCCM. Caregivers felt that CHWs were closer home, always available, and reliable. When a child was sick, caregivers cited how care could be accessed at any time of the day or night, and prompt. This is because CHWs were available and closer to them, especially when they did not have transport to hospital and during the night. Besides being closer, access to care was more reliable than services offered at formal health facilities since CHWs were always available, unlike in health centers, which might be closed due to limited operational hours, or have medicine stock-outs. Overall, caregivers expressed gratitude for the CHWs’ services, appreciating that CHWs were the first source of care they run to when children fall sick.

*“CHW is important because the child maybe falls sick at night and I do not have the means to take her to the hospital, the first person I’ll run to is CHW.” Caregiver_IDI20, Nambale.*

*“…everyone in our village has her number. … when a child falls sick at night and the hospital is closed, you just call so that she can see if its malaria, and if it’s not malaria, she sends you to Nambale if you can get there.” WRA_29, Nambale.*

The intervention also significantly improved access to appropriate treatment and follow-up. A higher proportion in the intervention area received ACTs (84.2% vs. 68.4%, *p* = 0.027), with CHWs being a major source (35.0% vs. 15.4%, *p* = 0.008), while private hospitals were a more significant treatment source in the comparison area (15.4% vs. 1.3%, *p* = 0.002). Furthermore, CHWs in the intervention area contributed significantly more to follow-up care (35.8% vs. 16.8%, *p* = 0.002) and the provision of malaria prevention advice (35.9% vs. 17.9%, *p* = 0.001). These findings demonstrate the effectiveness of CHW integration in enhancing community-level malaria care, from diagnosis to treatment and prevention.

The iCCM intervention in Nambale led to a significant decrease in pneumonia prevalence, dropping to 15.4% in the intervention site compared to 27.6% in the control site (*p* < 0.001). This reduction was accompanied by a notable change in initial care-seeking behavior, with a substantially higher proportion of caregivers in the intervention area first seeking care or advice from CHWs (18.5%) compared to the control area (3.7%, *p* = 0.035). Consequently, a significantly larger percentage of individuals in the intervention group sought care on the same day of symptom onset (55.8%) compared to the control group (35.2%, *p* = 0.031), indicating improved timeliness of care. Regarding treatment, while the overall proportion receiving treatment was similar, CHWs in the intervention area became a direct source of treatment for a higher percentage of individuals (7.5%) compared to none in the control group (*p* = 0.036). A higher proportion of the patients in the comparison group were treated at the health facilities (53%) compared to 40% from the intervention group. Furthermore, the use of antibiotic injections was exclusively observed in the intervention group (12.5% vs. 0.0%, *p* = 0.004).

In Tables 1–3, despite a statistically similar baseline prevalence of diarrhea (13.1% in the intervention vs. 17.4% in the comparison site, *p* = 0.124), the iCCM intervention significantly boosted care-seeking, with 73.4% of caregivers in the intervention area seeking advice or treatment compared to 53.9% in the comparison area (*p* = 0.049). A striking difference was the role of CHWs as the first point of contact, serving 25% of cases in the intervention area versus 0% in the comparison area (*p* = 0.002). This shift in care-seeking was accompanied by a significantly higher proportion of children in the intervention group receiving the recommended ORS and Zinc combination (73.5% vs. 54.0%).

**Table 3:** Pneumonia: care-seeking and access to appropriate treatment:

Measure		Intervention *n* = 382	Comparison *n* = 380	*p*-value
Prevalence of cough/ pneumonia		15.4% [*n* = 59]	27.6% [*n* = 105]	<0.001*
Sought treatment/advice for ARI		88.1%	78.1%	0.110
First source of treatment/advice for ARI	Government facility	42.3%	62.1%	0.024*
	Private Health facilities	1.9%	12.2%	0.03*
	Pharmacy	38.4%	25.2%	0.014*
	CHW	18.5%	3.7%	0.035*
Who received treatment		74.5%	75.2%	0.363
Treatment for ARI received	Antibiotic (amoxicillin)	62.5%	73.4%	0.221
	Antibiotic Injection	12.5%	0.0%	0.004*
	Cough syrup	32.5%	22.8%	0.254
	Panadol syrup	30.0	50.6	
Source of treatment	Public facilities	40.0%	43.0%	0.751
	Private facilities	0%	10.1%	0.033*
	Pharmacy	51.5%	54.4%	0.842
	Home remedies	2.5%	1.3%	0.561
	CHW	7.5%	0.0%	0.036*
Care sought within	Same day	55.8%	35.2%	0.031*
	Following day	19.2%	24.8	

The symbol * in the table indicates that the corresponding p-value is statistically significant at the 0.05 level (i.e., *p* < 0.05). This means the difference between the intervention and comparison groups for that particular measure is unlikely to have occurred by chance alone.

Furthermore, CHWs in the intervention area were the primary source of diarrhea treatment for a substantial 38.4% of cases, in contrast to 0% in the comparison area (*p* < 0.001). The qualitative findings corroborated this finding where the caregivers highlighted the important role of CHWs in providing treatment for diarrhea in children.

*“he had diarrhea. The CHW came, checked, and gave diarrhea tablets and ORS, and explained to me how to use them, …she also came by after a few days to see if he still had diarrhea and found he was okay.” Caregiver_IDI35, Nambale.*

*“… If the child has diarrhea, he gives you drugs…He gives you ORS. I went and gave him, and the diarrhoea stopped.” Caregiver_IDI31, Nambale.*

This direct involvement of CHWs also correlated with increased community engagement, as evidenced by significantly more CHW visits to children sick with diarrhea (30.6% vs. 7.9%, *p* = 0.003) and a greater provision of health education on diarrhea prevention within the last 3 months (23.3% vs. 10.2%, *p* < 0.001). Conversely, reliance on home remedies for diarrhea treatment was significantly higher in the comparison group (27.5%) compared to the intervention group (4.1%, *p* < 0.001) (Table 4).

**Table 4:** Diarrhea: care-seeking and access to effective treatment.

Measure		Intervention [*n* = 382]	Comparison [*n* = 380]	*p*-value
Diarrheal prevalence		13.1% [*n* = 49]	17.4% [*n* = 63]	0.124
Sought advice/ treatment for diarrhea		73.4%	53.9%	0.049*
First source of advice/ treatment for diarrhea	Public facilities	50.0%	67.6%	NS
	Private facilities	5.6%	8.8%	NS
	CHW	25%	0%	0.002*
	Pharmacy	16.7%	20.6%	NS
	Home remedies	0%	2.9%	NS
Received ORS/Zinc combination		73.5%	54.0%	NS
Source of diarrhea treatment	Government facilities	38.8%	41.7%	NS
	Private facilities	4.1%	7.8%	NS
	Pharmacy	14.3%	23.2%	NS
	Home remedies	4.1%	27.5%	<0.001*
	CHW	38.4%	0.0%	<0.001*
Care seeking within 24 h diarrhea		58.3%	41.4%	NS
CHW visit when child ill with diarrhea		30.6%	7.9%	0.003*
Health talk with CHW 3 months prevent diarrhea		23.3%	10.2%	<0.001

The symbol * in the table indicates that the corresponding p-value is statistically significant at the 0.05 level (i.e., *p* < 0.05). This means the difference between the intervention and comparison groups for that particular measure is unlikely to have occurred by chance alone.

### 3.3 CHWs’ experience of the digital health application and performance management approaches in conducting iCCM

#### 3.3.1 Positive experiences with the digital health application

##### 3.3.1.1 Improved efficiency and ease of use

CHWs frequently highlighted how the digital health application made service delivery faster and more convenient compared to manual methods. One CHW noted, *“The app makes work easy… I can finish within five minutes… But if I was doing it manually, I could use about fifteen minutes”* (CHW_IDI13, Nambale). The convenience of mobile entry reduced documentation burdens and saved valuable time during home visits.

##### 3.3.1.2 Clinical decision support and dosage accuracy

The application provided embedded clinical guidance, helping CHWs navigate complex care protocols with greater confidence. As one explained, *“When you do assessment, the phone directs you in everything. If the child has pneumonia, it gives dosage and is not easy to mess up… You tap the timer, it will direct and help you to count breaths”* (CHW_IDI24, Nambale). Another added, *“The phone helps by reminding me things I might have forgotten… like dosage according to age, and it just shows you the amount to give”* (CHW_IDI15, Nambale). These automated prompts ensured correct diagnosis and treatment, while minimizing human error. The app also reinforced referral protocols: *“If the patient needs to be referred, the phone will still tell you. If it is pneumonia, that phone has a timer that will help me count the breaths. The phone also reminds me to do follow ups”* (CHW_IDI23, Nambale).

##### 3.3.1.3 Built-in prompts and reminders

Beyond point-of-care guidance, the application actively prompted CHWs on essential visits and follow-ups. *“If there is a homestead that you have not visited, it will remind you. If you forgot to follow up, it will remind you. When a pregnant mother is almost getting to her delivery date, it will alert you. The phone is very useful. I would be crippled if I do not have it”* (CHW_IDI17, Nambale). Others shared similar experiences: *“On the task bar, it tells me… ‘Today you need to follow up this child.’ This woman is going to deliver tomorrow, she was supposed to finish her ANC. So this app reminds me”* (CHW_IDI13, Nambale). These digital nudges supported timely action and continuity of care, especially for time-sensitive maternal and child health services.

##### 3.3.1.4 Enhanced supervision and accountability

The digital platform allowed supervisors to remotely track CHWs’ field activities and data entry. *“Once you treat and put the data on, it will reach the LG office. So, they know who has gone to the field and who has not… If a week ends and you have not gone to the field, the dashboard will show”* (CHW_IDI17, Nambale). This functionality supported real-time supervision and follow-up: *“The phone is easy because once you have done your work and you sync and send, all your work remains in your phone after syncing and it is seen in the office”* (CHW_IDI27, Nambale). CHWs viewed this visibility as a motivator and accountability mechanism.

##### 3.3.1.5 Performance feedback and motivation

CHWs valued the ability to track their own performance via the app. *“The LG app is clear… It shows how many children I have attended to. How many I have treated—all the time, all the days—it shows you ‘Follow this one.’ Then you finish”* (CHW_IDI13, Nambale). Another explained, *“The phone shows you how you have done. You can press on the flag, press the keyboard, and there is a place where if you press, it shows you the total you have done for the whole month”* (CHW_IDI14, Nambale). This feedback loop reinforced a sense of accomplishment and encouraged sustained effort.

##### 3.3.1.6 Data security and reporting convenience

Digital reporting offered a safer and more reliable alternative to paper-based systems. *“Manual is hard because the MoH forms require that we write manually on the book and then take to them. What if you lose the register? With phone, if you sync the data, they will receive it”* (CHW_IDI18, Nambale). Others echoed this view: *“…with paper, sometimes you can write down your work and then it gets lost. But with phone, once you have sent it, it is safe. It cannot get lost”* (CHW_IDI25, Nambale). The ease of transmission and storage reduced the risk of lost records and improved efficiency: *“When you finish, you just send your data, and it gets to where it needs to go. We find that easier than these hard copies… with phone at least that information is safe”* (CHW_IDI24, Nambale).

#### 3.3.2 Challenges with the digital health application

##### 3.3.2.1 Technical glitches and data loss

Despite the advantages, several CHWs experienced system malfunctions that interfered with service delivery. *“Sometimes the phone goes off and tells me to follow up within 24 h. When I go back, my work has disappeared—and that is my incentive”* (CHW_IDI13, Nambale). Another reported, *“The app refuses sometimes. You may tap and it loads the whole day. By the time it starts working again, it has erased all your work… In October when my assessments had reached thirty, I only recovered six”* (CHW_IDI14, Nambale). These data losses were particularly demotivating, especially given that CHWs’ incentives are linked to submitted data.

##### 3.3.2.2 Connectivity and syncing delays

Application delays and syncing failures were commonly reported: *“Sometimes you can sync but it does not send data. Or if you submit your report, it goes round for long as it continues to sync… If it has not synced, you cannot treat another patient and input their information”* (CHW_IDI28, Nambale). This created frustration, especially in high-demand contexts where CHWs needed to move quickly between households.

##### 3.3.2.3 Power and charging constraints in rural areas

Inconsistent access to electricity posed a major barrier to optimal phone use. *“Charging is where there is a bit of a challenge because we are in the rural and we do not have electricity. To take it for charging, you need ten shillings, and you do not have”* (CHW_IDI21, Nambale). Others noted how lack of charging infrastructure delayed service delivery: *“I do not have electricity, so I have to take it for charging and they tell me to go back for this tomorrow since it is not yet full. So I lose time and miss a follow up”* (CHW_IDI13, Nambale).

## 4 Discussion

### 4.1 Care seeking behavior

The study findings demonstrate that although the overall proportion of caregivers seeking treatment for childhood illness was comparable between the intervention and comparison areas (80.0% vs. 71.6%), there were significant differences in the choice of healthcare provider. Utilization of CHWs was substantially higher in the intervention area, where 37.9% of caregivers sought care from CHWs compared to only 16.8% in the comparison area (*p* = 0.002). The increased utilization of CHWs in the intervention arm is likely attributable to several factors including: enhanced support performance management with active tracking of commodities and CHPs’ performance for target support supervision leading to consistent availability of medicines and supplies and improved case management competencies rippling to increased trust in CHWs driven by the digitalization platform. These findings align with existing literature, which indicates that performance management and supportive supervision can increase the community’s trust in CHWs and strengthen their role in primary healthcare delivery (16).

Similar overall treatment-seeking rates across both groups suggest that interventions did not necessarily increase caregivers’ behavior to seek care but rather changed their source of care toward trained CHWs within the community. This is a critical shift, as prompt and appropriate treatment at the community level is essential for child survival and health system efficiency. Additionally there was a greater reliance on private facilities in the comparison group (10.5% vs. 3.2%, *p* = NS). While the difference was not statistically significant (*p* = NS), the observed pattern could indicate a possible preference for private providers in the absence of strengthened community health services. This drift may reflect caregivers’ tendencies to bypass CHWs when they are unavailable, perceived as less effective, or inadequately supported (17).

### 4.2 Role of CHPs in iCCM

The findings of this study align with research conducted in other African countries, particularly regarding the vital role of CHWs in managing fevers at the community level. For example, Hamer et al. (18) in Zambia demonstrated how CHWs significantly contributed to malaria diagnosis and treatment in rural areas. The Zambian study also emphasized the effectiveness of home-based malaria management, which resonates with our findings reporting CHWs in the intervention group conducted a significantly higher number of malaria tests at the clients’ homes (45.1% vs. 16.9%, *p* < 0.001).

The findings reveal noteworthy differences in the patient care pathways between the intervention and comparison groups. In the intervention area, a smaller proportion of patients had their first point of care at health facilities (47%) compared to the comparison group (59%). Furthermore, only 41% of all patients in the intervention group ultimately received treatment at health facilities, whereas this figure reached 69% for malaria patients. In the intervention area, the reduced reliance on health facilities as the initial contact point is indicative of enhanced utilization and efficacy of community-based services, specifically those provided by CHWs. Studies have illustrated how critical it is to train and strengthen support supervision to CHPs for effective for improved consistency in community based integrated case management (19, 20). In concurrence with these studies, this study confirms that empowered and better supported CHWs through digital tools and strengthened supervision, increased community confidence in CHWs to manage common illnesses, potentially alleviating unnecessary referrals to higher-level care. The difference is particularly pronounced for malaria, with 69% of malaria patients in the comparison group requiring facility-based treatment versus only 41% in the intervention group. This likely reflects greater capacity among CHWs in the intervention area to diagnose and manage malaria at the community level, reducing the burden on primary health facilities. Early and effective malaria treatment administered by CHWs is critical for improving health outcomes and reducing morbidity and mortality, especially in rural or under-resourced contexts. The observed shift in care-seeking behavior aligns with prior research demonstrating that well-trained and supported CHWs can effectively manage uncomplicated cases, including malaria, at the community level (20).

### 4.3 Malaria management

CHWs played a pivotal role in facilitating home-based mRDT testing in the intervention area, with 45.1% of individuals tested at home compared to only 16.9% in the comparison group (*p* < 0.001). This aligns with findings that home-based testing improves accessibility and timeliness of care, particularly for populations in remote or underserved locations, and aligns with evidence that community-based mRDT delivery by CHWs leads to better targeting of antimalarial treatment and prompt management of febrile illnesses (21). This trend indicates that, in the absence of strengthened community systems, individuals may continue to rely on health facilities for diagnosis, potentially resulting in limited access for those facing geographical or economic barriers. The finding shows that a significantly higher proportion of individuals in the intervention area received ACTs (84.2% vs. 68.4%, *p* = 0.027), with CHWs serving as a major source of treatment (35.0% vs. 15.4%, *p* = 0.008), while private hospitals were more prominent treatment providers in the comparison area (15.4% vs. 1.3%, *p* = 0.002), underscores the effectiveness of community-based malaria case management strategies in improving access to timely and appropriate treatment. Importantly, these findings echo prior research by Kisia et al. (22) in Kenya found that CHWs played a crucial role in improving access to malaria testing and treatment. The higher ACT uptake in the intervention area reflects improved availability and utilization of standardized treatment at the community level, which is critical for reducing malaria morbidity and preventing.

In Uganda, Yeka et al. (23) observed that despite the presence of public health facilities, many people continued to rely on private clinics and pharmacies for malaria treatment. This trend was also evident in our study, where private facility use was higher in the comparison group (15.4% compared to 1.3% in the intervention). However, the Ugandan study highlighted inconsistencies in the quality of private-sector care, which may explain why the intervention group, supported by digitalization of CHWs operating under a robust performance management model, reported higher malaria case management by the CHWs compared to the comparison groups. These comparisons suggest that community-based interventions, when paired with digitalization and performance management remain a crucial strategy for improving malaria case management, particularly in rural and underserved populations.

Beyond malaria, this study also found a significantly lower prevalence of cough and pneumonia cases in the intervention group (15.4%) compared to the comparison group (27.6%, *p* < 0.001). This trend aligns with a systematic review by Gera et al., which examined various interventions aimed at reducing acute respiratory infections (ARI) in children under five. Additionally, the low prevalence of Pneumonia cases in the intervention group illustrates the impact of CHWs education to the community along prevention of Pneumonia cases even as they manage iCCM cases. Improved health education and caregiver awareness, common components associated with community-based interventions, may have contributed to increased preventive behaviors such as timely care-seeking, vaccination uptake, and household practices that reduce pneumonia risk. Higher treatment-seeking behavior in the intervention group (88.1%) compared to the comparison group (78.1%) suggests that CHWs may have played a key role in influencing health-seeking decisions. This surpasses figures reported by Goudarzi et al. (24), indicating the effectiveness of targeted interventions. The use of digital tools allowed CHWs to triage cases more accurately, ensuring timely management or referral and subsequent follow-up, mainly because of the technologically supported workflows promoted by Living Goods.

Differences in treatment sources were also evident, with the intervention group showing greater reliance on pharmacies (38.4%) and CHWs (18.5%). Greater utilization of pharmacies in the intervention area may indicate increased community trust and convenience associated with these outlets, which often serve as accessible first points of contact for health concerns due to extended hours, reduced waiting times, and geographic proximity. These patterns align with findings by Seneviratne et al. (25), who noted a growing reliance on pharmacies for primary healthcare. Additionally, a study by Reddy et al. (26) emphasized the crucial role of CHWs in improving health seeking behaviors, reinforcing our study’s findings. The preference for antibiotic injections in the intervention group (12.5%) versus none in the comparison group reflects broader treatment trends highlighted by Bhutta et al. (27). While antibiotics can be effective for bacterial infections, their overuse, especially for viral illnesses, raises concerns about antibiotic resistance. Notably, the intervention group had significantly higher same-day care-seeking rates (55.8% vs. 35.2%, *p* = 0.031), mirroring a study in Zimbabwe by Mbuya et al. (28), which found that timely interventions significantly improved ARI outcomes. This could be attributed to the accessibility of CHWs and knowledge among the community members on the range of health care services offered by the CHWs, hence ease of hailing CHWs for service delivery. In addition, digital alerts and automated client reminders may have played a role in enabling quicker follow-ups by the CHWs, as demonstrated in other settings where Living Goods’ digital performance tools are used.

### 4.4 Diarrhea management

Regarding diarrhea management, the intervention group demonstrated significantly better health-seeking behavior than the comparison group. Specifically, 73.4% of individuals sought treatment for diarrhea, compared to 53.9% in the comparison group. This indicates a significant improvement in health-seeking behavior attributable to the intervention. This enhanced treatment-seeking is a critical measure of community engagement and the effectiveness of strategies aimed at promoting timely and appropriate management of common childhood illnesses. The Improved health-seeking behavior in the intervention group may be linked to several factors embedded within the intervention framework, including increased awareness and education provided by CHWs, enhanced accessibility to community-based services, and strengthened performance management systems. This observation aligns with a 2020 study in Kenya by Amek et al. (29) which found that around 60% of caregivers sought treatment, though with notable variability. Notably, CHWs were the first source of advice for 25% of cases in the intervention group, compared to none in the comparison group. Additionally the finding mirrors a Tanzanian study by Mbuya et al. (30), where CHWs served as the first point of contact for 20% of caregivers.

Treatment patterns also differed significantly, with 73.5% of the intervention group receiving ORS/Zinc compared to 54.0% in the comparison group. This represents a significant improvement in adherence to recommended diarrhea management protocols. This enhanced uptake is critical because the combined use of ORS and Zinc constitutes the globally accepted standard treatment for acute childhood diarrhea. The higher prevalence of ORS/Zinc use in the intervention group likely reflects the positive influence of the intervention’s components such as community health worker training, digital performance management, and improved supply chain mechanisms. These factors may have increased caregiver awareness and access, ensuring that children with diarrhea receive timely, evidence-based treatment. These are similar to findings from Uganda. The intervention also led to a sharp decline in the use of home remedies (4.1% vs. 27.5%). and a significant increase in CHW reliance on treatment (38.4% vs. 0%). This reflects a significant behavioral shift toward evidence-based treatment approaches. This is likely attributable to enhanced community health education, improved access to formal healthcare services such as CHWs, and better availability of recommended therapies (e.g., ORS and zinc) promoted by the intervention. These trends mirror a 2020 Nigerian study by Abiola et al. (31) Similarly, while no significant difference was observed in care-seeking within 24 h (58.3% vs. 41.4%), these rates are comparable to a 2020 Ethiopian study by Tesfaye et al. where about 50% of caregivers sought care within the same timeframe.

Qualitative findings from caregivers underscored the proactive role of CHWs in promoting preventive measures and appropriate care-seeking for malaria. Caregivers highlighted the practical advice received from CHWs regarding environmental hygiene and consistent use of mosquito nets. This direct engagement by CHWs appears to contribute to the observed shift in initial care-seeking toward community-level providers, reducing reliance on more distant or costly private facilities, as evidenced by a greater reliance on private facilities in the comparison group (10.5% vs. 3.2%, *p* = NS). Moreover, the study noted consistently lower proportion of patients having their first point of care at the health facilities at 47% in the intervention group compared to 59% in the comparison group, with 41% being treated at the health facilities in the intervention group while 69% of the malaria patients received treatment at the health facilities. This aligns with broader literature suggesting that readily available community health services can significantly alter primary care access points and reduce burdens on higher-level facilities. Similar findings from Uganda have indicated challenges in malaria control, emphasizing the need for robust community-based strategies (23).

However, the qualitative data also revealed practical challenges that could impact the consistent provision of appropriate treatment, despite overall positive coverage statistics where a higher proportion in the intervention area received ACTs (84.2% vs. 68.4%, *p* = 0.027). Some caregivers expressed concerns about occasional stockouts of iCCM commodities. Addressing these supply chain issues is crucial for sustained effectiveness and ensuring that the positive shifts in care-seeking translate into consistent access to necessary medications. This is a common challenge in community health programs, as highlighted by various studies on supply chain management in low-resource settings, underscoring the need for robust logistics to support CHW effectiveness (31).

### 4.5 Pneumonia management

For pneumonia, the significant decrease in prevalence, dropping to 15.4% in the intervention site compared to 27.6% in the control site (*p* < 0.001), and the increased proportion of caregivers seeking initial care from CHWs (18.5% in intervention vs. 3.7% in control, *p* = 0.035), leading to more timely care (care sought on same day: 55.8% in intervention vs. 35.2% in control, *p* = 0.031), reaffirms that empowering CHWs can effectively reduce the burden on health facilities. The qualitative interviews further illuminated the critical role of CHWs in promoting positive care-seeking behaviors, particularly for maternal and child health, which extends beyond just acute illness management. Caregivers emphasized the consistent guidance and monitoring provided by CHWs throughout pregnancy and after childbirth.

### 4.6 Enablers and barriers to iCCM among CHWs

The qualitative findings underscore several critical enablers that supported the implementation of Integrated Community Case Management (iCCM) by Community Health Volunteers (CHWs), aligning with broader evidence on what drives successful community health programs. A key enabler reported by CHWs was the adequacy of training and technical support, which laid a strong foundation for effective service delivery. CHWs described receiving comprehensive initial training in iCCM protocols and regular refresher courses that strengthened their confidence and competence in managing childhood illnesses. This finding aligns with existing literature that emphasizes the importance of continuous capacity-building in improving the quality and consistency of CHW service delivery (32, 33).

Supportive supervision, particularly joint efforts by the Ministry of Health and implementing partners, was another commonly cited enabler. CHWs appreciated regular check-ins and field visits, which served not only as performance accountability mechanisms but also as motivational touch points that helped troubleshoot challenges and reinforce skills. This is consistent with broader evidence that structured and supportive supervision improves CHW retention, motivation, and adherence to clinical guidelines (34, 35). Another enabler was the use of performance-based incentives, which CHWs described as motivating and fair. Many noted that the target-based incentive model enhanced their sense of ownership and accountability. This blend of intrinsic motivation, derived from contributing to community health, and extrinsic rewards, such as financial incentives, mirrors findings from other studies showing that well-designed incentives improve CHW performance and reduce attrition (36, 37).

Digital health tools also emerged as a transformative enabler. CHWs reported that the mobile application used for service delivery and reporting streamlined their workflow, improved diagnostic accuracy, and provided timely prompts for follow-up care. These findings reinforce a growing body of evidence demonstrating how digitally enabled CHWs that are consistently supervised are more efficient and consistent (38, 39). The integration of such technologies into community health systems not only enhances service quality but also improves data visibility for health managers, supporting real-time decision-making and accountability.

Digital health interventions improved health outcomes and reduced the burden on health facilities. Systematic reviews showed these tools were generally cost-effective by enhancing efficiency, optimizing resource use, and improving patient care. However, variability in study methods highlighted the need for more standardized evaluations to accurately quantify their benefits (40). However, a few barriers were also evident. CHWs highlighted technical challenges such as app glitches and data syncing failures, as well as infrastructure limitations like lack of consistent electricity for phone charging. These issues occasionally led to data loss or delays in service provision, pointing to the need for stronger digital support systems and infrastructure investments to sustain gains made through digital health interventions (41).

## 5 Study limitations

Several limitations were experienced in this evaluation. First, the reported information on morbidity in the household survey is subjective in that it relied on the caregiver recall of illness and treatments provided. One important limitation of the study was the replacement of households that could not be located with the next available household within the same village. Even though this helped have the desired sample size and the practical implementation of the fieldwork, it may have introduced selection bias. Households that were replaced might have differed in socio-economic status, or health-seeking behavior—from those initially sampled potentially limiting the generalizability of results. Further, potential observation bias in the intervention group due to enhanced CHW support and the absence of a limitations section.

Lastly, a notable limitation of this study is the possibility that activities conducted by other partners in the Busia County and the surrounding may have impacted the control group. Due to this there is a reasonable likelihood that individuals in the control group experienced similar interventions through these external actors. Hence the measured effect of the digitalization and performance management strategies could be underestimated or confounded, making it more difficult to attribute observed changes solely to the intervention under investigation.

### 5.1 Public health implications

These findings emphasize the need for governments and health systems to invest in the sustainability and scalability of CHW programs for institutional support, integration with formal health structures, continuous training, supply chain strengthening, and financing mechanisms. These investments are important in achieving universal health coverage and health equity, particularly in low- and middle-income countries. The increased utilization of CHWs as source of care—evidenced by higher rates of treatment seeking, home-based malaria testing, and ACT administration—demonstrates how strengthening community health systems can improve timely access to effective, evidence-based interventions at the household level. This reduces the burden on health facilities, improves early case detection and management, and supports broader disease control efforts, which aligns with global evidence of CHWs’ contributions to reducing childhood morbidity and mortality, especially from malaria, pneumonia, and diarrhea.

The significantly higher CHW engagement and household visitation rates underscore the importance of effective performance management, supervision, and digital tools in motivating and enabling CHWs to maintain consistent community presence and service delivery. The observed shifts away from private and facility-based care toward accessible community-level providers suggest that well-supported CHW programs can enhance health system efficiency and affordability, particularly for vulnerable populations who face geographic and economic barriers.

## 6 Conclusion

Overall, these findings emphasize the critical role of CHWs in improving community health outcomes. Their involvement in malaria, pneumonia, and diarrhea management not only enhanced access to healthcare but also contributed to better access to first line commodities and more timely and consistent care-seeking behaviors. Additionally, there was consistently lower proportion of patients treated at the health facilities across all the iCCM cases in the intervention group compared to the comparison group, further highlighting the role of CHWs in managing the load at the health facilities in the community, leaving the facility staff to handle the severe cases. These results add to the growing body of evidence supporting community-based healthcare models as effective strategies for addressing common illnesses in resource-limited settings.

The findings of this study amplify the effectiveness of the iCCM strategy when integrated with digitalization and performance management in the communities. Driven by digitally enabled CHWs and data driven community health systems. This integration of digital tools with performance management practices strengthened CHW effectiveness, improved health outcomes, and contributed to the overall success of the iCCM strategy in the intervention area. At the heart of this success are CHWs, whose work not only increases the number of children under five receiving the right treatment for illnesses like pneumonia but also helps reduce reliance on traditional remedies for diarrhea. Interestingly the use of digital health app increased the efficiency of CHWs by refining their skills along iCCM management due to the guided workflows, facilitating timely reporting and tracking of coverage and service delivery hence informing decision making. Additionally, performance-based incentives provided extra motivation for the community health workers involved in the study.

The qualitative findings provide valuable lessons for scaling community-based interventions and guiding future maternal and child health policies and programs. The integration of digital health tools significantly improved community health worker efficiency and data reporting. Despite challenges like occasional commodity stockouts requiring continued attention, the overall evidence strongly supports the effectiveness of the iCCM model in strengthening community health services.

The iCCM program has played a vital role in helping families, especially in remote areas, seeking care when they need it most. By making essential child health services such as treatments for malaria, pneumonia, and diarrhea available right in the community, it has reduced the need for long journeys to distant health facilities and offered timely interventions. As a result, more children are receiving proper treatments in time, leading to fewer cases of serious illness and a notable decline in child deaths.

With CHWs actively testing and treating patients in the community, fewer cases end up at hospitals, which really eases the pressure on these health facilities. Additionally, CHWs are more appropriate in the iCCM intervention as they are from the community and can easily identify community challenges and promptly reach into poorest and hard-to-reach areas.

## Data Availability

The original contributions presented in the study are included in the article/supplementary material, further inquiries can be directed to the corresponding author/s.
